# β-Cell Obligation in α-Cell Glucagon Response to Low Glucose

**DOI:** 10.21203/rs.3.rs-9039050/v1

**Published:** 2026-04-15

**Authors:** Eva Bru-Tari, Marta Perez-Frances, Fabrizio Thorel, Pedro L. Herrera

**Affiliations:** Dept. of Genetic Medicine & Development, iGE3 and Centre facultaire du diabète, Faculty of Medicine, University of Geneva, 1206 Geneva, Switzerland

## Abstract

Type 1 diabetes (T1D) destroys insulin-producing pancreatic *β*-cells, causing chronic hyperglycemia. Patients are also vulnerable to life-threatening hypoglycemia, driven largely by defective *α*-cell glucagon secretion, yet the mechanisms underlying this counterregulatory failure remain poorly understood. Using human pseudoislets from non-diabetic donors and complementary mouse models, we show that *α*-cells isolated from all other islet cell types fail to mount counterregulatory glucagon responses to low glucose, fully recapitulating the diabetic phenotype. Strikingly, the presence of even a few *β*-cells per islet is sufficient to restore *α*-cell counterregulatory function. These findings demonstrate that hypoglycemia-induced glucagon secretion is not an intrinsic *α*-cell property but depends critically on *β*-cell input. Defective counterregulation in diabetes therefore reflects disrupted *α*-*β* cell communication rather than intrinsic *α*-cell failure. By identifying *β*-cell loss as the primary driver of impaired glucagon secretion, this work reframes counterregulatory dysfunction as an islet network defect and highlights restoration of intra-islet *β*-cell signaling as a therapeutic strategy for improving glycemic stability in diabetes.

Pancreatic islets are highly organized micro-organs in which α-, β- and δ-cells coordinate hormone secretion to maintain glucose homeostasis^1,2^. α-cells are central to counterregulatory defense against hypoglycemia, secreting glucagon when blood glucose falls while suppressing secretion at elevated glucose^3^. Despite decades of research, the mechanisms governing glucose-regulated glucagon secretion remain debated. The central unresolved question is whether α-cells sense and respond to glucose autonomously, or whether regulated glucagon secretion emerges from signals provided by neighboring islet cells^4,5^.

This question has direct clinical relevance. In type 1 diabetes (T1D), autoimmune destruction of β-cells^6^ is accompanied by profound α-cell dysfunction^7^. Although α-cells are largely preserved in the T1D pancreas^8–10^, patients exhibit severely impaired glucagon responses to hypoglycemia^11–15^, destabilizing glycemic control and increasing the risk of life-threatening hypoglycemic episodes^7,16^. In early type 2 diabetes (T2D), where β-cells are present but functionally impaired^17^, α-cell dysregulation manifest differently, as fasting and postprandial hyperglucagonemia^18^. However, in advanced or insulin-treated T2D, counterregulatory responses also become impaired^19,20^, suggesting that progressive β-cell failure converges on defects resembling T1D. Together, these observations implicate islet cellular context as a key determinant of α-cell behavior, and β-cell loss as a potential mechanistic driver.

Whether α-cell glucose responsiveness is cell-autonomous or network-dependent is therefore fundamental for understanding, and ultimately treating, α-cell dysfunction in diabetes. Using reconstituted human pseudoislets^21^ from non-diabetic donors with defined cellular composition, and mouse models with selective loss of specific islet cell types, we directly resolve this question, demonstrating that glucose-regulated glucagon secretion requires β-cell input.

## Glucose-regulated glucagon secretion in human α-cells requires intra-islet interactions

**Figure 1.**
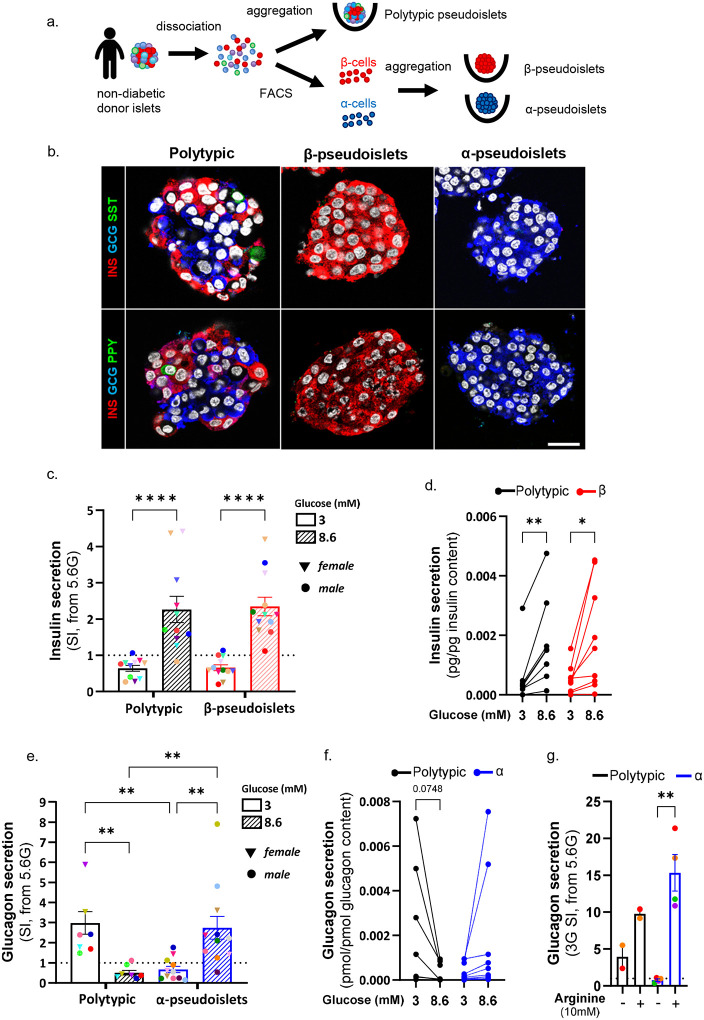
Glucose-regulated glucagon secretion is lost in human monotypic α-pseudoislets and requires intra-islet interactions. **a.** Generation of human pseudoislets. Islets from the same donor were dissociated and sorted by FACS to obtain highly pure α- and β-cells, which were reaggregated into monotypic pseudoislets. Control pseudoislets containing all endocrine cell types (“polytypic” pseudoislets) were generated by dissociating and reaggregating intact islets without sorting. **b.** Immunofluorescence staining of control pseudoislets (Polytypic), monotypic β- and α-pseudoislets. Upper panel: INS: red; GCG: blue; SST: green; lower panel: INS: red; GCG: blue; PPY: green. Scale bar: 20μm. **c.** *In vitro* insulin secretion expressed as stimulatory index (SI; fold change over basal glucose, 5.6 mM) at hypoglycemic-like (3 mM) and postpradial-like (8.6 mM) glucose concentrations in polytypic (black) and monotypic β-pseudoislets (red). n=11 donors (polytypic), n=12 donors (β-pseudoislets). **d.** Insulin secretion from (c) normalized to total insulin content. n=8 (polytypic), n=9 (β-pseudoislets). **e.** *In vitro* glucagon secretion expressed as SI at hypoglycemic-like (3 mM) and postpradial-like (8.6 mM) glucose in polytypic (black) and monotypic α-pseudoislets (blue). n=7 polytypic, n=10 α-pseudoislets **f.** *G*lucagon secretion from (e) normalized to total glucagon content. n=8 (polytypic), n=9 (α-pseudoislets). **g.** *G*lucagon secretion expressed as SI at hypoglycemic-like glucose (3 mM) ± arginine (10 nM) stimulation in all cells (black) and monotypic α-pseudoislets (blue). n=2 (polytypic), n=4 (α-pseudoislets). 1-way ANOVA with Sidak’s multiple comparisons test (c, e); paired t-test within pseudoislet type (d, f, g). Samples from the same donor are color-coded across conditions (c, e, g) or connected by a line across pseudoislet type (d, f). All data are shown as mean±sem. Raw data are provided as Source Data Fig.1.

To determine whether human α-cells regulate glucagon secretion autonomously, we examined their secretory behavior in isolation from other islet-cell types. We generated three-dimensional human islet-like aggregates (“pseudoislets”) from non-diabetic donors, as either “polytypic” pseudoislets containing all major islet cell types or “monotypic” pseudoislets composed exclusively of purified α- or β-cells isolated by fluorescence-activated cell sorting (FACS)^21^ (Fig. 1a–b, **Suppl. Fig.1a-b**).

Pseudoislets were exposed to basal (5.6 mM), then either hypoglycemic (3 mM) or postprandial (8.6 mM) glucose concentrations to assess direction-specific secretory responses within physiological ranges. Polytypic pseudoislets displayed appropriate hormone secretion: insulin increased at 8.6 mM and decreased at 3 mM (stimulatory index, SI>1 and <1, respectively; Fig. 1c). Monotypic β-pseudoislets recapitulated this profile (Fig. 1c–d; **Suppl. Fig. 1c**), confirming that β-cells retain cell-autonomous glucose-responsiveness in isolation^22^.

Polytypic pseudoislets showed physiological glucagon regulation, with secretion increasing at 3 mM and suppressed at 8.6 mM (SI>1 and <1, respectively; Fig. 1e). In stark contrast, α-pseudoislets failed to suppress glucagon release at 8.6 mM (SI≥1) and exhibited a markedly blunted counterregulatory response at low glucose (Fig. 1e–f). Critically, α-pseudoislets contained glucagon levels comparable to polytypic pseudoislets (**Suppl. Fig. 1d**) and responded robustly to arginine stimulation (Fig. 1g), confirming preserved secretory capacity and a selective defect in glucose-regulated, rather than generalized, glucagon secretion.

These results demonstrate that human α-cells fail to mount appropriate glucose-dependent responses in isolation, revealing that proper glucagon regulation requires interactions with other islet cell types. Strikingly, the dysregulated glucagon profile closely mirrors that observed in intact islets from individuals with T1D^23–25^, pancreatic slices^26^ and plasma across glycemic ranges (**Suppl. Fig. 2a**)^11–15,27,28^, who display both blunted counterregulatory responses to hypoglycemia and impaired suppression at elevated glucose. These findings directly implicate disrupted intra-islet signaling, rather than intrinsic α-cell failure, as the mechanism underlying defective glucagon responses in T1D.

## Adjacent β-cells restore glucose-regulated glucagon secretion in human α-cells

Given that α-cells display aberrant glucagon secretion in isolation, and that residual β-cell mass in T1D, reflected by circulating C-peptide, correlates with partial preservation of counterregulatory responses^13,29^ (**Suppl. Fig. 2a**), we hypothesized that β-cells provide signals essential for α-cell glucose responsiveness.

**Figure 2.**
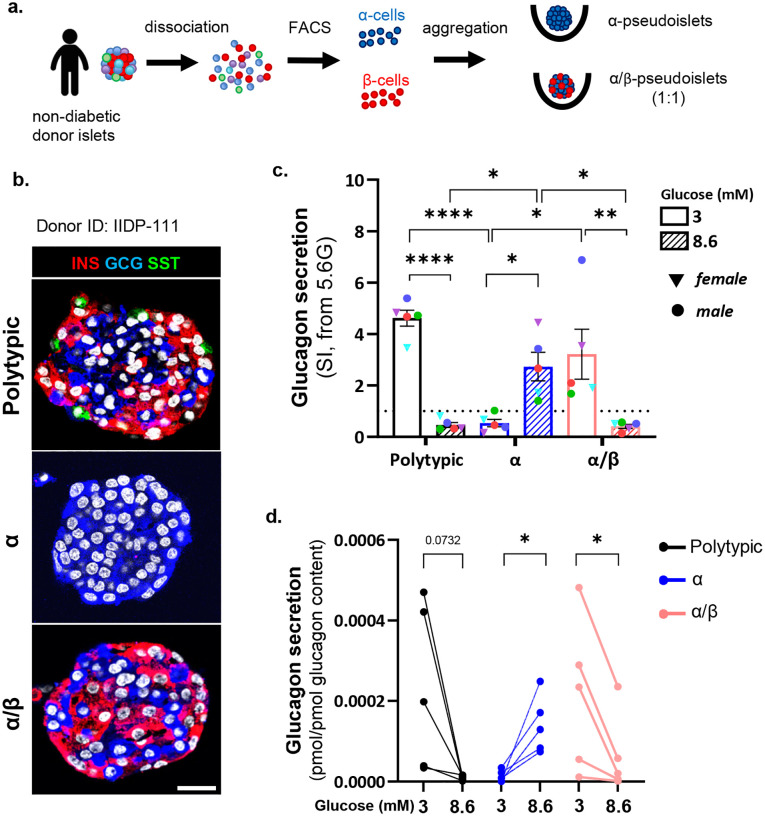
β-cell presence is sufficient to maintain glucose-regulated glucagon secretion in human α-cells. **a.** Generation of human pseudoislets. Islets from the same donor were dissociated and sorted by FACS to obtain highly pure α- and β-cells, which were reaggregated into monotypic α-pseudoislets and mixed αβ-pseudoislets. Control pseudoislets containing by all endocrine cell types (“polytypic” pseudoislets) were generated by dissociating and reaggregating intact islets without sorting. **b.** Immunofluorescence staining of control pseudoislets (polytypic), monotypic α-pseudoislets and mixed αβ-pseudoislets. INS: red; GCG: blue; SST: green. Scale bar: 20μm. **c.** *In vitro* glucagon secretion expressed as SI at hypoglycemic-like (3 mM) and postpradial-like (8.6 mM) glucose in polytypic (black), monotypic α-pseudoislets (blue) and αβ-pseudoislets (light-red). **d.** *G*lucagon secretion from (c) normalized to total glucagon content. N=5 donors. Samples from the same donor are color-coded across conditions (c) or connected by a line across pseudoislet type (d). 2-way ANOVA with Tukey’s multiple comparisons test (c), paired t-test within pseudoislet type (d). All data are shown as mean±sem. Raw data are provided as Source Data Figure 2.

To test this directly, we generated binary human pseudoislets composed exclusively of α-and β-cells (“αβ pseudoislets”). FACS-purified α-cells were re-aggregated with purified β-cells at a 1:1 ratio, approximating their relative abundance in native human islets, ensuring close physical proximity within the same three-dimensional structure (i.e. the pseudoislet) (Fig 2a–b, **Suppl. Fig.1b**). Glucagon secretion was assessed as above and compared with monotypic α-pseudoislets and polytypic pseudoislets from the same donors.

αβ-pseudoislets exhibited robust glucagon release at 3 mM glucose and effective suppression at 8.6 mM, closely recapitulating the response of polytypic pseudoislets (Fig. 2c–d). Insulin and glucagon content in αβ-pseudoislets were comparable to polytypic pseudoislets and monotypic α-only and αβ-pseudoislets, respectively (**Supp. Fig. 1e-f**), confirming that restoration of glucose-regulated glucagon secretion was not attributable to differences in glucagon abundance.

These findings directly refute a cell-autonomous model of α-cell glucose responsiveness and demonstrate that β-cell proximity is sufficient to maintain or restore physiological glucagon regulation. They further imply that the profound β-cell loss in T1D is itself sufficient to drive aberrant glucagon secretion, independently of other disease-related factors, establishing β-cells as essential regulators of α-cell function.

## β-cell loss abolishes glucose-regulated glucagon secretion *in vivo*

**Figure 3.**
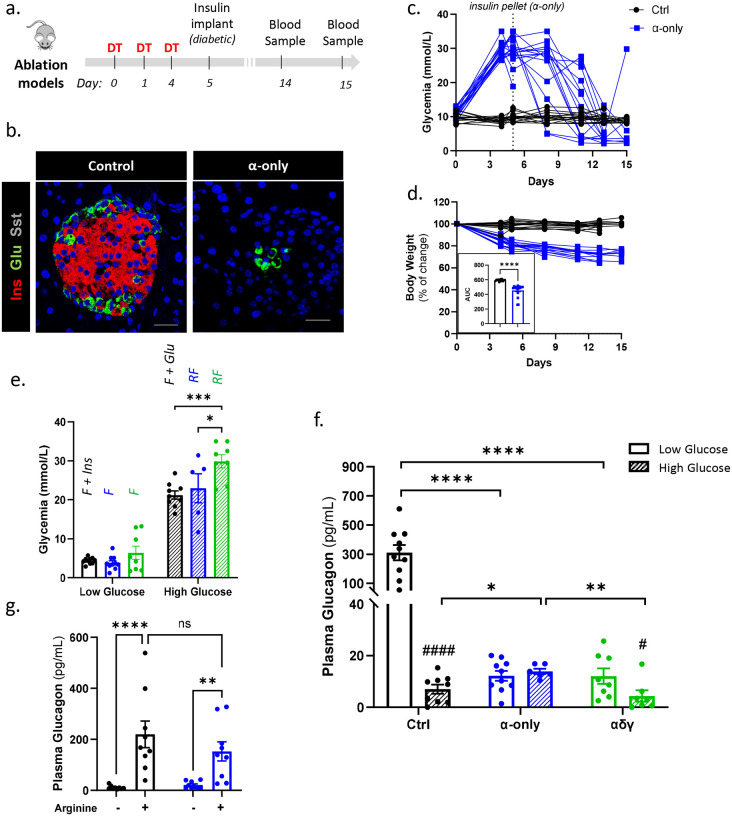
β-cell loss abolishes α-cell counterregulatory glucagon secretion in vivo. **a.** Experimental design. Any mouse model of cell ablation is injected with diphtheria toxin (DT, three doses of 120ng) and implanted with insulin pellets one day after the last DT injection. Blood samples in hyper and hypoglycemia are collected at day 14 and 15 after the first DT injection, respectively. **b.** Immunofluorescence staining of control and “α” mice. INS: red; GCG: green; SST: gray. Scale bar: 20μm. **c.** Glycemia over 15 days after first DT injection in control (Ctrl, black, n=16) and “α” (blue, n=17) mice. **d.** Percentage of body weight change in control (Ctrl, n=16) and “α” (blue, n=17) mice. Inset: Area under the curve (AUC) of body weight change. Two-tailed Mann-Whitney test **e.** Glycemia of control (black, low glucose: n=10; high glucose: n=9), “α” (blue, low glucose: n=10; high glucose: n=5) and “αδγ” (blue, n=8) mice before blood collection at low (dashed bar) and high (fill bar) glucose levels. Mice were fasted (F) or random fed (RF) and injected with intra-peritoneal insulin (Ins) or glucose (Glu) to achieve comparable glucose levels between groups. **f.** Plasma glucagon (pg/ml) in control (Ctrl, black, low glucose: n=10; high glucose: n=9), “α” (blue, blue, low glucose: n=10; high glucose: n=6) and “αδγ” (green, low glucose: n=8; high glucose: n=7) at low (dashed bar) and high (continuous bar) glucose levels. Statistical comparisons between groups in at each glucose condition were performed using one-way ANOVA with Tukey’s post-hoc test (*). Comparisons between low vs high glucose within each group were performed using two-tailed Mann Whitney test (#). **g.** Glucagon secretion (pg/ml) at low glucose levels before and after arginine (10nM) stimulation in control (black, n=9) and “α” (blue, n=9) mice. Twoway ANOVA with Fisher’s test (*). All data are shown as mean±sem. Raw data are provided as Source data Figure 3.

The pseudoislet experiments establish that α-cell glucose responsiveness depends on β-cell interactions. To determine whether this network dependence operates *in vivo*, we examined glucagon secretion in transgenic mouse models with defined islet endocrine cell compositions. Using diphtheria toxin (DT)-mediated selective cell ablation, we generated mice with islets composed exclusively of α-cells (“α-only”) or lacking β-cells while retaining α-, δ- and γ-cells (“αδγ” mice) (Fig. 3a–b; **Suppl. Fig. 3a**).

Within four days of DT administration, both β-cell-deficient models developed severe hyperglycemia and significant weight loss (Fig. 3b–d; **Suppl. Fig. 3b-c**). Exogenous insulin was administered to prevent lethality. Despite treatment, mice exhibited marked glycemic instability under *ad libitum* feeding, with frequent hypoglycemic excursions mirroring the hypoglycemia susceptibility observed in T1D (Fig. 3c; **Suppl. Fig. 3b**).

To assess α-cell glucose responsiveness *in vivo*, plasma glucagon was measured under both hypoglycemic and hyperglycemic conditions. Hypoglycemia was induced by 5-hour fasting in “α-only” and “αδγ” mice; controls required fasting plus insulin administration to reach comparable low glucose levels (Fig. 3e; **Suppl. Fig. 3d**). Both “α-only” and “αδγ” mice displayed markedly blunted plasma glucagon responses under hypoglycemia, in stark contrast to the robust counterregulatory rise mounted by controls (Fig. 3f). Importantly, “α-only” mice retained robust glucagon secretion in response to arginine (Fig. 3g), confirming preserved secretory capacity and a selective loss of glucose-dependent regulation.

“α-only” mice exhibited reduced pancreatic glucagon content (**Suppl. Fig. 4a-b**), consistent with loss of PPY-expressing cells that co-express glucagon^30^. To exclude reduced α-cell mass as a confounding factor, we examined “αγ” mice, which retain α- and γ-cells and maintain normal pancreatic glucagon content (**Suppl. Fig. 4a-b**). These mice exhibited similarly blunted counterregulatory responses, hypoglycemic episodes, and weight loss as “α-only” mice (**Suppl. Fig. 4c-f**), confirming that impaired counterregulation reflects loss of β-cell input rather than reduced α-cell mass.

Under hyperglycemic conditions, controls showed significantly higher glucagon at low versus high glucose, reflecting robust dynamic regulation (Fig. 3f). “α-only” mice exhibited nearly identical glucagon levels across both glycemic states, indicating complete loss of glucose responsiveness. “αδγ” mice showed only a modest reduction in glucagon at high glucose (Fig. 3f), suggesting that δ-cell somatostatin contributes to glucagon suppression at elevated glucose but is insufficient to restore counterregulatory responses in the absence of β-cells.

Together, these findings demonstrate that glucose-dependent glucagon secretion in vivo is not cell-autonomous. β-cell loss alone abolishes α-cell counterregulatory responses during hypoglycemia, recapitulating the hallmark glucagon defect of T1D and corroborating the human pseudoislet findings.

## A minimal β-cell mass is sufficient to maintain α-cell counterregulation

**Figure 4.**
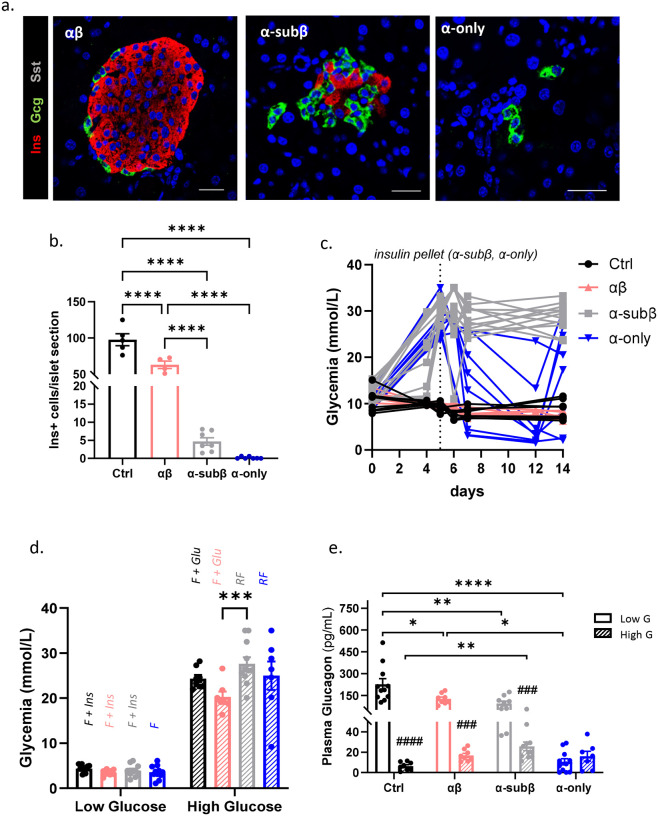
A minimal β-cell population is sufficient to ensure α-cell counterregulatory function in vivo. **a.** Immunofluorescence staining of “αβ”, “α” mice treated with suboptimal DT dose “α subβ” and “α”. INS: red; GCG: green; SST: gray. Scale bar: 20μm. **b.** Quantification of insulin+ cells per islet section in control (Ctrl, black, n=7), “αβ” (pink, n=4), “α subβ” (grey, n=7) and “α” (blue, n=7). **c.** Glycemia over 15 days after first DT injection in control (Ctrl, black, n=8), “αβ” (pink, n=6), “α subβ” (grey, n=12) and “α” (blue, n=8). **d.** Glycemia of control (black, low glucose n=11; high glucose n=8), “αβ” (pink, low glucose n=8; high glucose n=7), “α subβ” (grey, low glucose n=9; high glucose n=11) and “α” (blue, low glucose n=11; high glucose n=7) before blood collection at low (dashed bar) and high (fill bar) glucose levels. Mice were fasted (F) or random fed (RF) and injected with intra-peritoneal insulin (Ins) or glucose (Glu) to achieve comparable glucose levels between groups. **e.** Plasma glucagon (pg/ml) in control (black, low glucose n=11; high glucose n=8), “αβ” (pink, n=8), “α subβ” (grey, low glucose n=9; high glucose n=11) and “α” (blue, low glucose n=11; high glucose n=7) at low (dashed bar) and high (continuous bar) glucose levels. Statistical comparisons between groups at each glucose condition were performed using one-way ANOVA with Tukey’s post-hoc test (*). Comparisons between low vs high glucose within each group were performed using two-tailed Mann Whitney test (#). One way ANOVA with multiple comparisons test (b, e). All data are shown as mean±sem. Raw data are provided as Source data Figure 4.

We next asked whether a residual β-cell population is sufficient to sustain α-cell counterregulatory function *in vivo*. We generated mice with islets composed exclusively of α- and β-cells (“αβ” mice) or retaining a residual β-cell population alongside α-cells (“α-subβ” mice), achieved by administering suboptimal doses of DT to “α-only” mice, to preserve an average of 4.7±0.9 β-cells per islet section while effectively ablating δ- and γ-cells (Fig. 4a, b). β-cell numbers in “αβ” mice were slightly reduced relative to controls, likely reflecting the ablation of bihormonal insulin–pancreatic polypeptide co-expressing cells^30^. These groups were compared to “α-only” mice, in which high-dose DT reduced β-cells to an average of 0.2±0.1 per islet section.

All β-cell-deficient diabetic mice (“α-only” and “α-subβ”) required exogenous insulin to manage post-ablation hyperglycemia. Strikingly, spontaneous hypoglycemic excursions under *ad libitum* feeding occurred exclusively in “α-only” mice (Fig. 4c, also Fig. 3c), whereas “α-subβ” mice were protected from hypoglycemia despite receiving comparable insulin treatment (Fig. 4c). This directly attributes hypoglycemic susceptibility to β-cell loss rather than insulin treatment.

To directly assess α-cell counterregulatory capacity, plasma glucagon was measured during hypoglycemia (Fig. 4d). All groups retaining β-cells (controls, “αβ” and “α-subβ” mice), mounted robust glucagon responses at equivalent low glucose levels, in stark contrast to the counterregulatory failure of “α-only” mice (Fig. 4e, see also Fig. 3f). Although glucagon responses in “αβ” and “α-subβ” mice were slightly below control levels, they were clearly distinguishable from “α-only” mice, and greater β-cell abundance correlated with stronger counterregulatory capacity (Fig. 4e). Under hyperglycemic conditions, controls, “αβ”, and “α-subβ” mice all showed significantly higher glucagon at low versus high glucose, reflecting intact glucose-dependent regulation, whereas “α-only” mice again lacked this dynamic range (Fig. 4d–e).

Collectively, these *in vivo* findings demonstrate that glucose-regulated glucagon secretion is not a cell-autonomous property of α-cells but an emergent function of the islet network requiring β-cell input (**Suppl. Fig. 2b**). Critically, even a minimal β-cell population is sufficient to sustain counterregulatory glucagon release, consistent with clinical observations that residual β-cell mass in T1D partially preserves counterregulation^13,29^ (**Suppl. Fig. 2a**). The α-β interdependence revealed here underscores the clinical importance of preserving residual β-cell mass in T1D to maintain counterregulatory responses and reduce the risk of severe hypoglycemia.

## Discussion

Involving the integration of metabolic, electrical and paracrine inputs^31^, glucose-regulated insulin secretion persist in β-cells even in complete isolation^22^. Here, we show that α-cells are fundamentally different: glucose-regulated glucagon secretion requires β-cells input. Using human pseudoislets and complementary mouse models, these findings resolve a long-standing debate over α-cell autonomy^4,32^, reframe counterregulatory glucagon release as an emergent property of the islet network and implicate disrupted intercellular signaling, rather than intrinsic cellular failure, as the primary driver of α-cell dysfunction in diabetes.

Prevailing models hold that α-cells intrinsically encode counterregulatory responses to hypoglycemia^33,34^, with paracrine signals from β- and δ-cells shaping suppression at high glucose^35,36^. However, prior evidence relied on intact islets, where intra-islet communication persists, or dispersed cells lacking three-dimensional architecture. Using monotypic human α-pseudoislets and mice with α-only islets, we show that α-cells cannot independently generate their canonical glucagon response. In both species, glucagon secretion during hypoglycemia is impaired, resembling a T1D. Notably, arginine-stimulated glucagon release remains intact in both models, indicating a preserved secretory competence and a selective defect in glucose-regulated, rather than generalized, glucagon secretion. Minor species differences (modest excess *ex vivo* secretion at high glucose in human α-cells versus uniformly low glucagon across *in vivo* glycemic conditions in “α-only” mice), likely reflect experimental context differences.

A central finding is that β-cells are essential for α-cell glucose responsiveness. In both species lacking β-cells, α-cells failed to respond to low glucose. Strikingly, a minimal β-cell presence, as in binary human αβ-pseudoislets or “αβ” and “α-subβ” mouse models, was sufficient to preserve counterregulatory glucagon secretion. This cross-species observation underscores a fundamental α-β interdependence consistent with native islet architecture, in which human α-cells almost universally co-reside with β-cells^37^, and extensive α-β juxtaposition is enforced by the classical core-mantle organization^38^.

These findings argue against intrinsic α-cell failure as the cause of defective counterregulation in T1D^25,26,39–43^. α-cells from non-diabetic donors function normally within polytypic or “αβ”-pseudoislets, yet reproduce a T1D-like glucagon secretion pattern when deprived of non-α-cellular context in monotypic α-pseudoislets. Disrupted intercellular signaling, not intrinsic α-cell incompetence, therefore underlies the hallmark glucagon defects of T1D. Beyond the inflammatory stress, loss of α-β communication emerges as a primary driver of impaired α-cell counterregulation in T1D patients with advanced β-cell loss. The same logic applies to T2D, where progressive β-cell dysfunction likely disrupts this communicative axis. Critically, the presence of residual β-cells in T1D patients may be sufficient to therapeutically preserve counterregulatory responses.

Excessive δ-cell somatostatin release is proposed to cause impaired α-cell counterregulation in T1D^44^, and somatostatin receptor antagonism partially restores glucagon responses in patients^45^. However, in the absence of β-cells, removing δ-cell input cannot restore hypoglycemia-induced glucagon secretion, as α-cells remain unresponsive in models lacking both β- and δ-cells. Therefore, somatostatin excess amplifies, rather than initiates, defective counterregulation after β-cell loss.

Beyond regulating glucagon secretion, β-cells actively constrain α-cell identity^46^. β-cell ablation or interrupted insulin signaling triggers spontaneous α-to-β-cell conversion in mice^47,48^, a process enhanced by forced expression of β-cell transcription factors^48^. In human α-pseudoislets spontaneous cellular conversion is not observed^21^. Instead, human α-cells adopt a β-cell-like stimulus-secretion profile, with high glucose inducing rather than suppressing secretion, suggesting a transition to a new functional state rather than simple loss of regulation, which highlights the potential for plasticity seen in forced reprogramming^21^. β-cells thus serve a dual role: maintaining glucose-regulated glucagon secretion while restraining α-cell reprogramming^48^. Residual β-cell mass in diabetes may therefore critically influence the efficiency of α-cell conversion-based regenerative strategies.

In summary, we show that glucose-regulated glucagon secretion is not an intrinsic α-cell property, but a network-dependent process dictated by β-cell input. Because β-cell loss alone mimics the counterregulatory defects of T1D, disrupted α–β communication emerges as the central mechanism underlying impaired glucagon responses. Restoring islet intercellular signaling represents a compelling therapeutic strategy for improving glycemic stability in diabetes.

## METHODS

### Human islets and pseudoislet generation

All studies involving human samples were approved by ethical committee in University of Geneva. Human pancreatic islets were obtained from the Geneva University Hospital, the NIDDK-funded Integrated Islets Distribution Program (IIDP) at City of Hope and from the University of Alberta Diabetes Institute Islet Core (Edmonton, Alberta, Canada). Subject details are described in Extended Data Table 1.

Upon arrival, islets were cultured overnight in fresh CMRL 1066 media (11530–037, Gibco) at 37°C and 5% CO_2_. Dissociation of human islets and staining with cell-surface antibodies to purify α- and β-cells was performed as described previously^,21,22,49^. Stained cells were sorted on a Moflo Astrios (Beckman Coulter) system. Single viable islet cells were gated by forward scatter, side scatter and pulse-width parameters and by negative staining for DAPI (D1306, Invitrogen) to remove doublets and dead cells. For pseudoislets formation, agarose structures with spherical microwells were obtained employing a micro-mold (3D Petri Dish, Microtissues Inc., Providence RI, USA) and then used to promote self-assembly of the cells in a density of 1,000 cells/pseudoislet. Every other day culture media was changed. Experiments were performed 7–10 days after culture, when pseudoislets were strongly aggregated. Pseudoislet morphology and cell composition were evaluated by immunostaining and RT-qPCR as detailed below.

### RNA extraction and RT-qPCR

Human pseudoislets were frozen in RLT buffer (Qiagen) with β-Mercaptoethanol and stored at −80 °C before being processed for RNA extraction using the Qiagen RNeasy Micro Kit. cDNA of human pseudoislets was generated using the Qiagen QuantiTect Reverse Transcription Kit. RT-qPCR reactions were performed using the appropriate primers mixes for each gene as well as the Express SyBr© GreenER kit (Invitrogen #100001652). We used the CorbettRobotics4 robot, and the PCR reaction was completed in the CorbettResearch6000 series cycler using a 40 cycles program. Biological duplicates of each sample were run in triplicate. Expression levels were normalized to *RN18S*. Primers sequences are shown in Extended Data Table 2.

### Human pseudoislets *in vitro* static glucose-stimulated insulin and glucagon secretion

Human pseudoislets were handpicked for each assay replicate and washed by incubation for 1 hour at 37°C in secretion medium containing 2.5mM CaCl2, 140mM NaCl, 4.5mM KCl, 1mM MgCl2, 20mM Hepes and 5.6 mM glucose (Sigma) in dH2O. Samples were then transferred into fresh secretion medium containing 5.6 mM (basal) glucose for 1 hour followed by incubation for 30 minutes in secretion medium containing 8.6 mM (postprandial-like) glucose or 3 mM (hypoglycemic-like) glucose at 37°C. 10 mM Arginine (Sigma, 11009) supplementation in the secretion medium containing 8.6 mM (postprandial-like) glucose was added to assess the response of α-cells to a known stimuli. Medium was collected after incubation at each glucose concentration and stored at −80°C for subsequent analysis. Pseudoislets were recovered and lysed for total insulin/glucagon content using acid ethanol (74% ethanol, 1.4% HCl). Insulin concentration was measured using the Insulin Chemiluminiscence ELISA (ALPCO, 80-INSHU-CH01). Glucagon concentration was analyzed by ELISA (Mercodia, 10–1271-01).

### Animals

To generate the different mouse models used in this study, the previously described Sst-DTR^22^, Gcg-DTR^50^, Ppy-DTR^30^ and Rip-DTR^47^ transgenes were combined depending on the cell ablation strategy. Mice were housed in closed cages with density varying depending on the size of the cage, in accordance with the Swiss regulation (Cage type S to L, Charles River). Cages were enriched with bedding, nestlet and a mouse house. Temperature and humidity in the housing room was maintained between 20–24 °C and 30–70%, respectively. The day-night cycles were programmed by alternating 12 h day–12 h night. Animals received food and tap water ad libitum. Male mice were used for experiments. Animals were randomly allocated to control or treatment groups. The study follows all ethical regulations regarding animal experimentation, all experiments were performed under the guidelines of the Direction General de la Santé du Canton de Genève (license numbers: GE15820 and GE359A).

### Diphtheria toxin administration

Diphtheria toxin (DT, Sigma) was injected intra-peritoneally on days 0, 1 and 4 to two-month-old mice. The dose of DT varied depending on the experiment: 126 ng of DT per injection (“α-only, “αδγ”, “αβ” and “αγ” mice) or 5ng of DT per injection (“α subβ” mice). Mice received insulin pellet subcutaneously (Linbit) upon hyperglycemia (>25mM).

### Blood collection at hypo- and hyperglycemia

For measurements at low blood glucose, diabetic mice with susceptibility to hypoglycemia (“α-only”, “αδγ”, “αγ” mice) were subjected to 5 hours fasting; while controls, “αβ” and “α-subβ” mice were intraperitoneal injected with insulin (0.5 U/kg Actrapid, Novo Nordisk A/S, Bagsværd, Denmark) after 5 hours of fasting to achieve comparable hypoglycemia levels. Blood from the tail vein was collected before and 30 minutes after the challenge. For measurements at hyperglycemia, blood from diabetic mice (“α-only”, “αδγ”, “αγ” and “α-subβ” mice) was collected in the random-fed state during the morning; whereas non-diabetic mice (controls and “αβ” mice) received an intraperitoneal injection of 20% glucose solution (relative to their body weight) following 5 hours of fasting. Blood from the tail vein was collected before and 15 minutes after glucose administration.

Aprotinin (Sigma; A6279) was added to the blood samples to avoid glucagon degradation.

### Arginine test

Mice were fasted for 2h before starting the experiments. A 20% L-arginine (Sigma; 11009) solution was administered intraperitoneal relative to their body weight. Glycemia was measured before the injection, 15 and 30min after arginine administration. Blood from the tail vein was collected before and 15min after arginine injection. Aprotinin (Sigma; A6279) was added to the blood samples to avoid glucagon degradation.

### Tissue protein extracts

Pancreatic tissue was homogenized in acid-ethanol solution (74% ethanol, 1.4% HCl), then sonicated and centrifuged. The supernatant was collected and used for immunoassay experiments, which were performed following the manufacturer’s instructions (Mercodia Ultrasensitive Mouse Insulin ELISA 10–1249-01; Mercodia Glucagon ELISA 10–1281-01).

### Plasma hormones measurement

Insulin (Mercodia Ultrasensitive Mouse Insulin ELISA, 10–1249-01), Glucagon (Mercodia Glucagon ELISA, 10–1281-01) were measured in plasma samples obtained from blood extracts in the tail vein. All analyses were performed following the manufacturer’s instructions.

### Immunofluorescence

Mouse pancreas paraffin sections were 5 μm thick and human pseudoislet cryosections were 10 μm thick. The primary antibodies used were: rabbit anti-insulin (1:3000; Molecular Probes, 701265), chicken anti-insulin (1:200; Abcam ab14042); mouse anti-glucagon (1:1000; Sigma, G2654), rabbit anti-glucagon (1:200; DAKO, A0565), rabbit anti-somatostatin (1:200; DAKO, A0566), rat anti-somatostatin (1:200; Merck Mab354), mouse anti-Ppy (1:400; Santa Cruz, MAB62971). Sections were also stained with DAPI. Secondary antibodies were coupled to Alexa 488, 405, 568, 647 (1:500; Molecular Probes) or TRITC (1:500; Southern Biotech). All sections were examined with a confocal microscope (Leica TCS SPE).

### Meta-analysis of plasma glucagon dynamics across the glucose range

We performed a focused compilation of published human studies reporting plasma glucagon and concurrent glucose measurements. Studies were included if they compared non-diabetic participants with either T1D, or T1D subjects with different levels of residual C-peptide, and employed experimental designs such as mixed-meal tests, oral glucose tolerance test, insulin infusions or hyperinsulinemic-hypoglycemic clamps. Studies lacking quantitative glucagon data were excluded. When numerical values were not provided in the text or tables, glucagon and glucose means were extracted from published figures using WebPlotDigitizer (version 5). A total of 9 studies^11–15,27–29,51^ were included and are summarized in Source data Supplemental Figure 2a.

Glucagon concentrations were converted to pmol/L and glucose values to mM. Mean glucagon values were normalized to each study’s basal glycemia to yield fold-change responses. Basal glucagon levels of each study were reported as fold change = 1. Glucagon-glucose pairs were pooled across studies for analyses. Glucose-dependent glucagon responses were modeled according to physiological plausibility and goodness-of-fit criteria. Non-diabetic subjects and T1D with detectable C-peptide showed glucose-dependent release of glucagon and were therefore fitted using a four-parameter logistic (4PL) model. T1D subjects without residual C-peptide lacked sigmoidal behavior and were analyzed using linear regression, reflecting their monotonic or absent normal glucose-glucagon correlation. Curve comparisons were performed using the extra sum-of-squares F-test, with goodness-of-fit assessed by R^2^. Analyses were conducted in GraphPad Prism 9.0, and data each point in Supplemental figure 2 represents a study-level mean. All data were derived from previously published sources.

### Statistics and reproducibility

Data are presented as mean ± SEM, as indicated in the figure legends. Statistical analyses were performed using Prism v9.0 software. Paired t-test, Mann–Whitney two-sided tests, one- or two-way ANOVA were employed for comparison as indicated in the figure legends (ns *p*>0.05; *p≤0.05; **p≤0.01; ***p≤0.001; ****p≤0.0001). P-values are described each figure legend and Source Data Tables. The significant statistical differences are indicated in the figures. More than three mice per condition and experiment were analyzed as indicated in the figure legends and Source Data Tables. Immunofluorescence was performed more than once for each mouse with >4 paraffin-sections/mouse.

## Supplementary Files

This is a list of supplementary les associated with this preprint. Click to download.

- Supplementalfigures.pdf
- ExtendedDataTables12.pdf
